# Cysteine cathepsin C: a novel potential biomarker for the diagnosis and prognosis of glioma

**DOI:** 10.1186/s12935-021-02417-6

**Published:** 2022-02-02

**Authors:** Xingbo Cheng, Zhishuai Ren, Zhendong Liu, Xiang Sun, Rongjun Qian, Chen Cao, Binfeng Liu, Jialin Wang, Hongbo Wang, Yuqi Guo, Yanzheng Gao

**Affiliations:** 1 Department of Surgery of Spine and Spinal Cord, Henan Provincial People’s Hospital, People’s Hospital of Zhengzhou University, People’s Hospital of Henan University, No.7, Weiwu Road, Henan 450003 Zhengzhou, China; 2 People’s Hospital of Zhengzhou University, Henan Provincial People’s Hospital, Zhengzhou, Henan China; 3 School of Basic Medical Science, Xinxiang Medical University, Xinxiang, Henan China; 4 Department of Neurosurgery, Henan Provincial People’s Hospital, People’s Hospital of Zhengzhou University, People’s Hospital of Henan University, Zhengzhou, Henan China; 5 Henan University People’s Hospital, Henan Provincial People’s Hospital, Zhengzhou, Henan China; 6 Department of Obstetrics and Gynecology, Henan Provincial People’s Hospital, People’s Hospital of Zhengzhou University, People’s Hospital of Henan University, No.7, Weiwu Road, Zhengzhou, Henan 450003 China; 7 Henan International Joint Laboratory for Gynecological Oncology and Nanomedicine, Zhengzhou, Henan China

**Keywords:** Glioma, Cysteine cathepsin C, Biomarker, Gene set enrichment, Prognosis, Piperlongumine, scopoletin

## Abstract

**Background:**

Cysteine cathepsin C encoded by the *CTSC* gene is an important member of the cysteine cathepsin family that plays a key role regulation of many types of tumors. However, whether *CTSC* is involved in the pathological process of glioma has not yet been reported. We comprehensively analyzed data from multiple databases and for the first time revealed a role and specific mechanism of action of *CTSC* in glioma, identifying it as a novel and efficient biomarker for the diagnosis and treatment of this brain tumor.

**Methods:**

The expression of *CTSC* in glioma and its relationship with clinical characteristics and prognosis of patients with glioma were analyzed at different levels by using clinical sample information from several databases. *CTSC* expression levels in glioma and normal brain tissues, as well as in glioma cells and normal brain cells, was validated by real-time quantitative polymerase chain reaction (RT-qPCR). Gene set enrichment analysis (GSEA) was used to reveal the signaling pathways that *CTSC* may participate in. The connectivity map was used to reveal small molecules that may inhibit *CTSC* expression in glioma, and the putative effect of these compounds was verified by RT-qPCR.

**Results:**

Our analyses showed that the expression of *CTSC* in glioma was higher than that in non-cancerous cells. GSEA showed that *CTSC* expression may regulate the malignant development of glioma through Toll-like receptor signaling pathways, pathways in cancer, and extracellular matrix receptor interaction signaling pathways. And we proved piperlongumine and scopoletin could inhibit *CTSC* expression in glioma cells.

**Conclusions:**

*CTSC* may serve as an efficient molecular target for the diagnosis and therapy of glioma, thereby improving the poor prognosis of patients with glioma.

**Supplementary Information:**

The online version contains supplementary material available at 10.1186/s12935-021-02417-6.

## Introduction

Glioma is one of the most common primary malignant tumors of the central nervous system. There are approximately 200,000 new cases of glioma worldwide each year, and the fatality rate is also extremely high, which poses a heavy burden on society and families [1]. At present, the 5 year survival rate of patients with glioma is less than 10%, which is much lower compared to some other cancers [2]. The reasons for the low survival rate may be related to the complex pathological mechanism and biological characteristics of glioma, such as strong self-renewal ability, high invasiveness, strong angiogenesis, and high recurrence rate [3, 4]. In recent years, many studies have shown that the lack of effective targets for the diagnosis, treatment, and prognosis evaluation is one of the bottlenecks in the treatment of glioma [5]. Therefore, searching for specific and effective biomarkers for the diagnosis, treatment, and prognosis evaluation of glioma has been an important and challenging topic in recent years.

According to the report of the National Comprehensive Cancer Network, many potential glioma biomarkers have been identified and utilized, including mutated isocitrate dehydrogenase (IDH) and O-6-methylguanine-DNA methyltransferase (*MGMT*) genes, 1p/19q co-deletion status, *BRAF* fusion, and CpG island methylator phenotype [6, 7]. Among them, *MGMT* encodes a DNA repair protein that rapidly repairs DNA alkylation damage caused by alkylating agents and maintains genomic stability in cells. *MGMT* is involved in the development of resistance to alkylating chemotherapeutic drugs in glioma, and its promoter methylation has been considered as a potential biomarker for the outcome of patients with glioma [8, 9]. In the World Health Organization (WHO) 2016 guidelines, the 1p/19q co-deletion status and *IDH* mutation were used as important bases for glioma classification [10]. However, because glioma has complex pathological mechanisms involving many factors, simple biomarkers cannot play a decisive role in the diagnosis and prognosis of this brain tumor [11]. Therefore, identification and investigation of more efficient biomarkers are urgently needed.

Cathepsins include many cysteine, serine, and aspartate proteases [12]. The cysteine cathepsin family is composed of 11 proteases that have different conformations and catalytic activities, as well as distinct distribution patterns and physiological functions in tissues and cells [13]. Cathepsins regulate the invasion, apoptosis, and drug resistance of malignant tumors [14, 15]. Recent studies have found that cysteine cathepsins B, S, L, K, and X are abnormally overexpressed in glioblastoma, and their levels are closely related to the extent of glioblastoma malignancy [13, 16–19]. Cysteine cathepsin C (encoded by *CTSC*), known also as dipeptidyl peptidase I, is one of the members of the cysteine cathepsin family. Previous studies have reported that the *CTSC* gene is overexpressed in many cancers. For example, Khaket et al. revealed that *CTSC* mediates the proliferation of colorectal cancer cells by regulating autophagy, which plays a key role in the tumor microenvironment and tumor development [20, 21]. Additionally, Zhang et al. showed that the expression of *CTSC* in hepatocellular carcinoma is increased and that *CTSC*, as an oncogene, promotes the malignant progression of this cancer through the interaction with the TNF-α/p38 MAPK signaling pathway [22]. Moreover, *CTSC* has been shown to control the infiltration of immune cells in the skin tumors and promote angiogenesis in squamous cell carcinoma [23]. Based on the above studies of the relationship between *CTSC* expression and the occurrence and development of various cancers, we believe that *CTSC* can be used as a carcinogenic gene marker, as it is related to the adverse progression of many cancers. The latest report has confirmed that aging-related genes do have an impact on glioma, including *CTSC* [24]. The innovation of our article is to mainly focus on *CTSC*, and study in detail from multiple levels about its effect on glioma survival and prognosis, and try to reveal the underlying mechanism.

In this study, we investigated the clinical significance of the *CTSC* expression pattern in gliomas of different grades. In addition, we predicted the potential mechanism by which *CTSC* may promote malignant glioma progression by using the gene set enrichment analysis (GSEA). Moreover, we screened and verified two small-molecule drugs that inhibited *CTSC* expression, which may expand the treatment of glioma. Our results suggest that *CTSC* may be a novel prognostic marker and a potential antitumor target for the treatment of glioma.

## Materials and methods

### Data collection

The GEPIA (http://gepia.cancer-pku.cn/index.html) database is a public data platform that integrates current cancer genomics data from The Cancer Genome Atlas (TCGA) database and sequencing results of normal human tissues from Genotype-Tissue Expression Project (GTEx) (https://www.genome.gov/Funded-Programs-Projects/Genotype-Tissue-Expression-Project). The database is convenient for rapid visualization of gene expression in tumor and normal tissues, and the gene expression profile data can be dynamically analyzed for single gene analysis, cancer species analysis, polygenic analysis [25]. In this study, we used the GEPIA database to determine the level of *CTSC* expression in glioma samples comparing with normal brain tissues. A total of 163 glioblastoma multiforme (GBM) samples and 518 low-grade glioma samples from TCGA database, and 207 normal brain tissue samples from GTEx were included.

The GEO database (https://www.ncbi.nlm.nih.gov/geo/) is a free and open gene expression database created and maintained by the National Center for Biotechnology (NCBI). The GEO database contains high-throughput gene expression data submitted by research institutions worldwide [26]. We obtained three datasets from the GEO database to analyze the expression of *CTSC* in glioma, namely GSE4290, GSE15824, and GSE50161. Due to the differences in the included samples of different research teams, we only selected the gene expression profiles in the samples of high grade glioma and normal brain tissue for analysis, including 77 glioblastoma grade IV samples and 23 normal brain tissue samples in GSE4290, 12 primary GBM samples and 2 normal brain tissue samples in GSE15824, and 34 GBM samples and 13 normal brain tissue samples in GSE50161.

The HPA database (https://www.proteinatlas.org/) is a publicly available database containing transcriptomic and proteomic expression data from different human tissues and organs at the RNA and protein levels [27]. Based on the HPA database, we analyzed CTSC expression in different brain samples at the protein level.

The Chinese Glioma Genome Atlas (CGGA) database (http://www.cgga.org.cn/) contains functional genomic data of approximately 2,000 Chinese glioma samples and detailed clinical data, including patient sex, age, radiotherapy and chemotherapy status, as well as complete follow-up data. In total, 748 CGGA RNA-seq glioma samples (218 WHO grade II glioma, 240 WHO grade III glioma, and 290 WHO grade IV glioma) and 268 CGGA microarray glioma samples (100 WHO grade II glioma, 52 WHO grade III glioma, and 116 WHO grade IV glioma) were selected from the database, and the clinical information of the corresponding patients is listed in Additional file 1: Tables S1 and S2.

The Cancer Genome Atlas (TCGA) (https://portal.gdc.cancer.gov/) is the largest cancer gene information database. TCGA covers 33 cancer types and more than 30,000 tumor samples, and it includes multiple omics data, such as gene expression data, miRNA expression data, copy number variation, and DNA methylation data. In total, 653 TCGA RNA-seq glioma samples (238 WHO grade II glioma, 256 WHO grade III glioma, and 159 WHO grade IV glioma) were selected from the database, and the clinical information of the corresponding patients is listed in Additional file 1: Table S3.

### Gene set enrichment analysis

Gene set enrichment analysis (GSEA) is a calculation method that determines whether a predefined gene set shows significant consistency differences between two biological states [28]. In our study, the generated gene sequence was used as a predefined gene set based on the correlation of *CTSC* expression, and the GSEA method was then used to reveal the significant survival difference between the groups with high and low *CTSC* expression. NES > 1.8, *P* < 0.05 and FDR < 0.25 indicate statistical significance.

### Connectivity Map database analysis

The Connectivity Map (CMap) database (https://portals.broadinstitute.org/cmap/) integrates genome-wide transcriptional expression data from cultured human cells exposed to various small-molecule drugs and has analytical tools to enable interrogation of the relationships between drugs, genes, and diseases. The database stores information about the effects of thousands of drugs and has data for 10,000 cell lines [29]. Based on the 748 glioma samples in the CGGA RNA-seq database, we used Pearson’s correlation to obtain 20 genes whose expression co-varied with *CTSC* expression, including 10 positively related genes and 10 negatively related genes. Then, we loaded these genes as upregulated and downregulated genes, respectively, into the CMap database to obtain several small-molecule compounds that could have inhibitory effects on the expression of *CTSC* using the following criteria: enrichment > 0.8 and *P* < 0.05. Finally, we obtained the molecular formulae, PubChem CID, two-dimensional structure, and three-dimensional structure of these small molecules from PubChem (https://pubchem.ncbi.nlm.nih.gov/). In addition, we verified the effect of these small-molecule compounds on the expression of *CTSC* in glioma cells by RT-qPCR.

### Glioma cell culture and preparation of glioma specimens

Glioma cell lines LN-229, T98, A172, and human astrocyte (HA) cell line were provided by the Microbiology Laboratory of the Henan Provincial People’s Hospital. The cells were cultured in high glucose Dulbecco′s Modified Eagle′s Medium (Procell, China) supplemented with 10% fetal bovine serum (Gibco, US) and 1% penicillin-streptomycin mixture at 37 °C in a humidified incubator in the atmosphere of 95% air and 5% carbon dioxide. Twenty-three glioma samples and nine non-glioma samples were rapidly frozen in liquid nitrogen within 15 min after surgical resection, and all patients with glioma were diagnosed by qualified pathologists. All patients provided written informed consent in accordance with the principles of the Declaration of Helsinki. The study protocol was approved by the Ethics Committee of the Henan Provincial People’s Hospital (2020, Ethical Review No. 107).

### RNA isolation and RT-qPCR

Total RNA was extracted using Trizol (Invitrogen, US) according to the manufacturer’s instructions, and cDNA was obtained using NovoScript Plus All-in-one 1st Strand cDNA Synthesis SuperMix (gDNA Purge) (Novoprotein, China). RT-qPCR was performed using NovoStart SYBR qPCR SuperMix Plus (Novoprotein, China). RNA-specific primer sequences for the internal reference gene *18 S* were as follows: forward, 5′-GTAACCCGTTGAACCCCATT-3′ and reverse, 5′-CCATCCAATCGGTAGTAGCG-3′. The specific primer sequences of the target gene *CTSC* were as follows: forward, 5′-TCAACTGCTCGGTTATGGGA-3′ and reverse, 5′-GTAAATGATGGTGAAATGGC-3′.

### Statistical analysis

All data in this study were analyzed using R software (v0.3.6.1 version). The Wilcoxon or Kruskal–Wallis tests were used to determine the correlation between *CTSC* and clinical characteristics of patients with glioma. The Kaplan–Meier and Cox analyses were used to reveal the impact of *CTSC* expression level on the prognosis of patients with glioma and whether *CTSC* had reliable diagnostic value for glioma prognosis. The univariate Cox and multivariate Cox analyses were used to determine whether *CTSC* was an independent risk factor for glioma. The Pearson’s correlation analysis was used to identify genes that were co-expressed with *CTSC*. Statistical significance was set at *P* < 0.05. In GSEA, effects were considered statistically significant if the normalized enrichment score > 1.8, *P* < 0.05, and false discovery rate < 0.25.

## Results

### Elevated expression of *CTSC* in glioma and other tumors

**Fig. 1:**
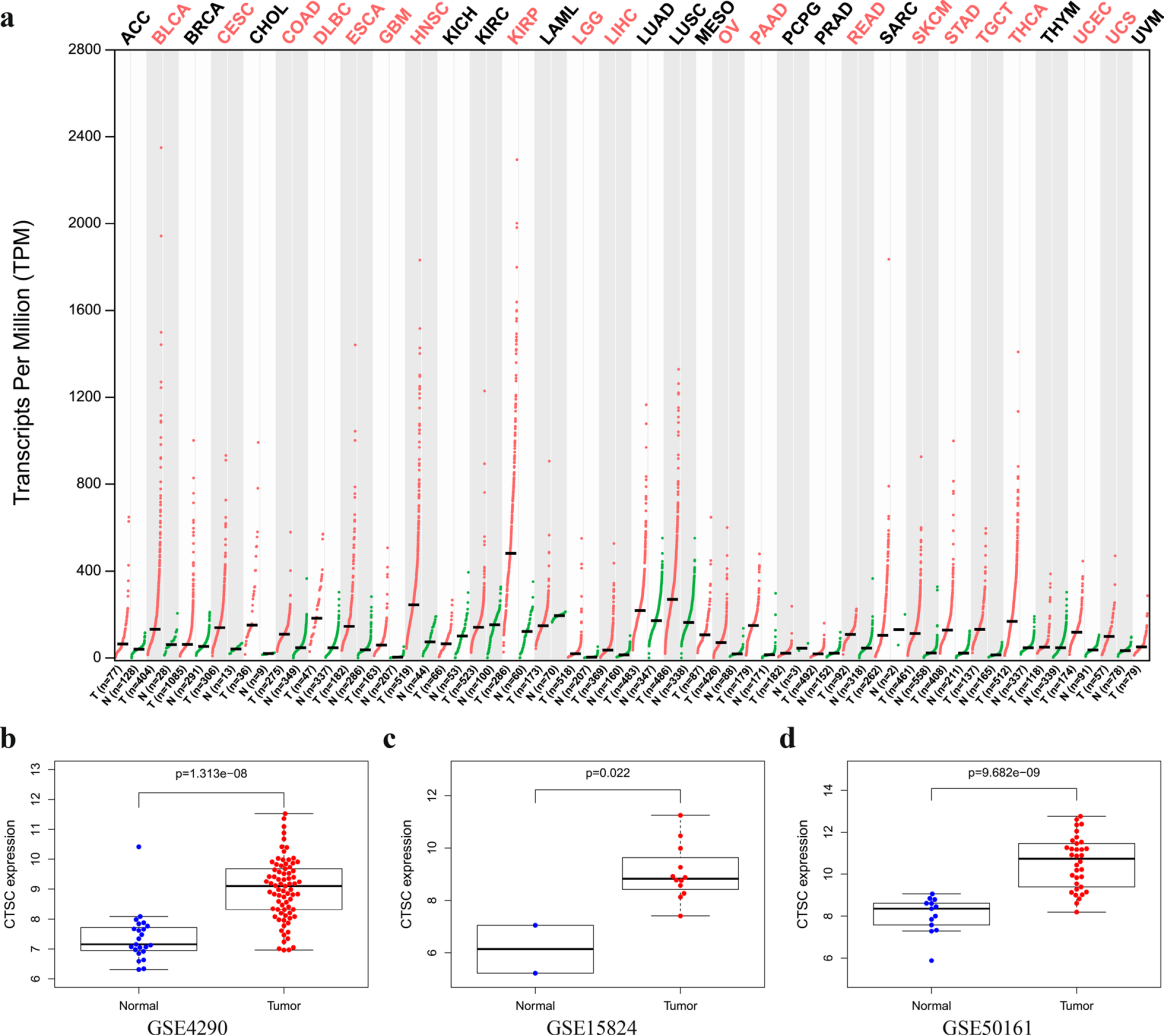
Expression of *CTSC* in glioma based on the GEPIA and the GEO database. **a** *CTSC* is abnormally overexpressed in a variety of tumors including GBM and LGG.** b**–**d** *CTSC* is abnormally overexpressed in glioma based on GSE4290, GSE15824, and GSE50161 from the GEO database

**Fig. 2:**
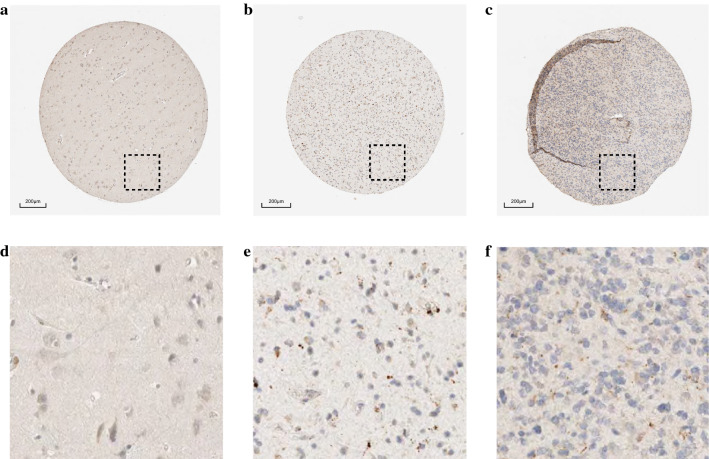
Expression of CTSC in glioma based on the HPA database. **a** Normal brain tissue. **b** Low grade glioma. **c** high grade glioma.** d**–**f** they represent the local enlarged drawings of** a**–**c** respectively

We explored the expression pattern of *CTSC* in human tumors based on the GEPIA database and found that *CTSC* was highly expressed in many tumors (red marked), including GBM and low-grade glioma (Fig. 1a). Further, the analysis of multiple datasets in the GEO database also showed that *CTSC* was overexpressed at the molecular level in glioma (Fig. 1b–d). To verify the evidence of increased *CTSC* mRNA levels in tumors in these databases, we further evaluated the expression of *CTSC* at the protein level based on the samples in the HPA database. We found that CTSC protein expression in glioma samples was higher than that in normal brain tissues. Moreover, the protein expression of CTSC in high-grade glioma was higher than that in low-grade glioma (Fig. 2).

**Fig. 3:**
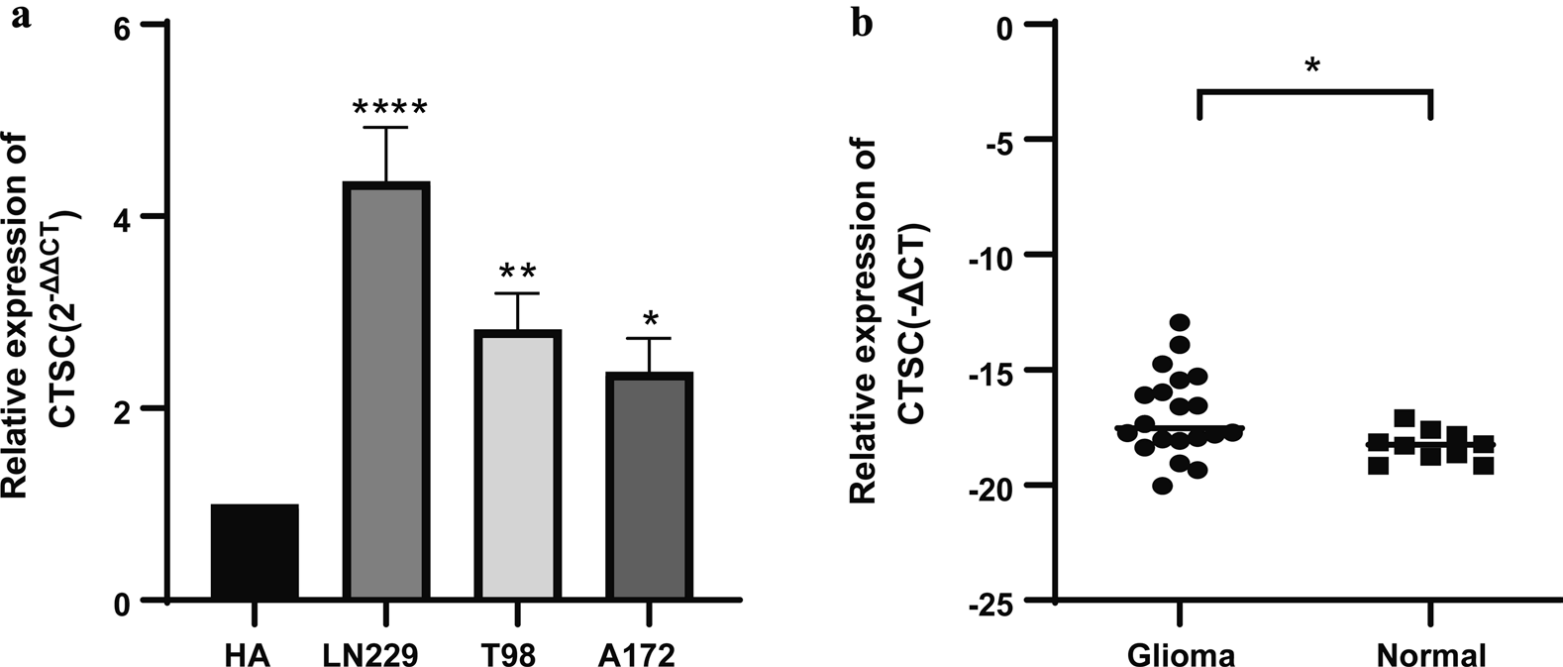
The expression of *CTSC* was increased in both glioma cell lines and glioma tissues based on RT-qPCR. **a** The expression of *CTSC* was increased in glioma cell lines. **b** The expression of *CTSC* was increased in glioma tissues

Next, we verified the *CTSC* expression level in clinical glioma samples and laboratory glioma cell lines by RT-qPCR. We found that compared with its level in normal human astrocytes, the *CTSC* expression level in glioma cell lines was significantly (>twofold) higher, with the highest, fourfold increase being observed in LN-229 cells (*P* < 0.05, Fig. 3a). In the clinical brain tissue samples collected by us, the expression of *CTSC* in 23 patients with glioma was higher than that in 9 normal brain tissue samples (*P* < 0.05, Fig. 3b).

### High ***CTSC*** expression is associated with poor prognosis in patients with glioma

**Fig. 4:**
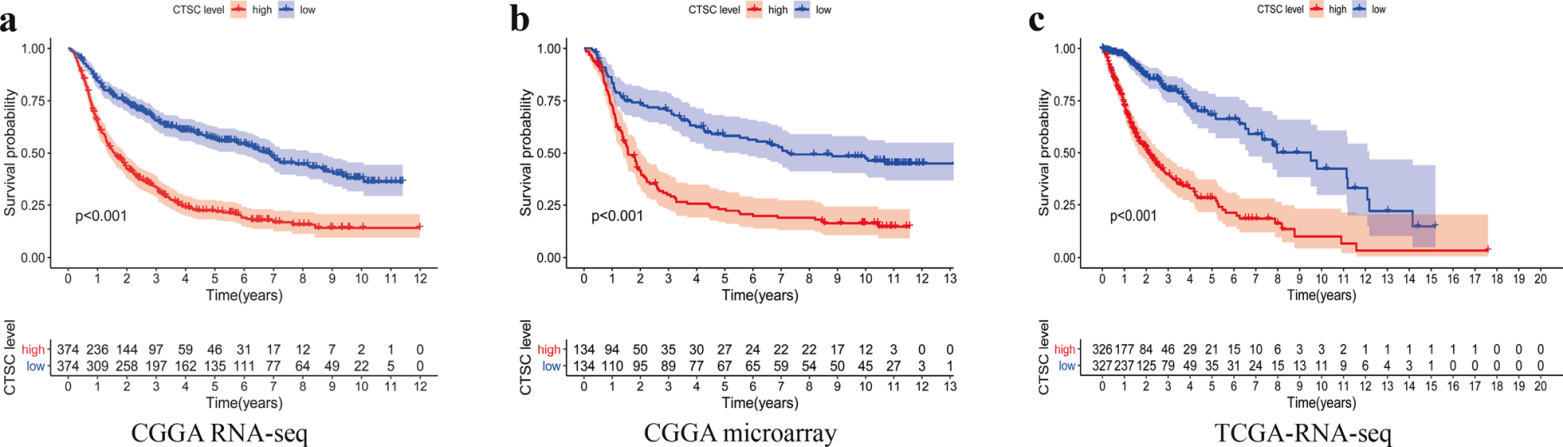
The abnormal high expression of *CTSC* is associated with the poor prognosis of glioma patients from three databases. **a** CGGA RNA-seq database. **b** CGGA microarray database. **c** TCGA RNA-seq database

To explore whether the level of *CTSC* expression is associated with the prognosis of patients with glioma, we analyzed clinical samples from three databases, namely, CGGA RNA-seq, CGGA microarray, and TCGA RNA-seq. We found that the survival of patients with glioma that had high *CTSC* expression was shorter than of patients with low *CTSC* expression (*P* < 0.001, Fig. 4).

### Receiver operating characteristic curve analysis

**Fig. 5:**
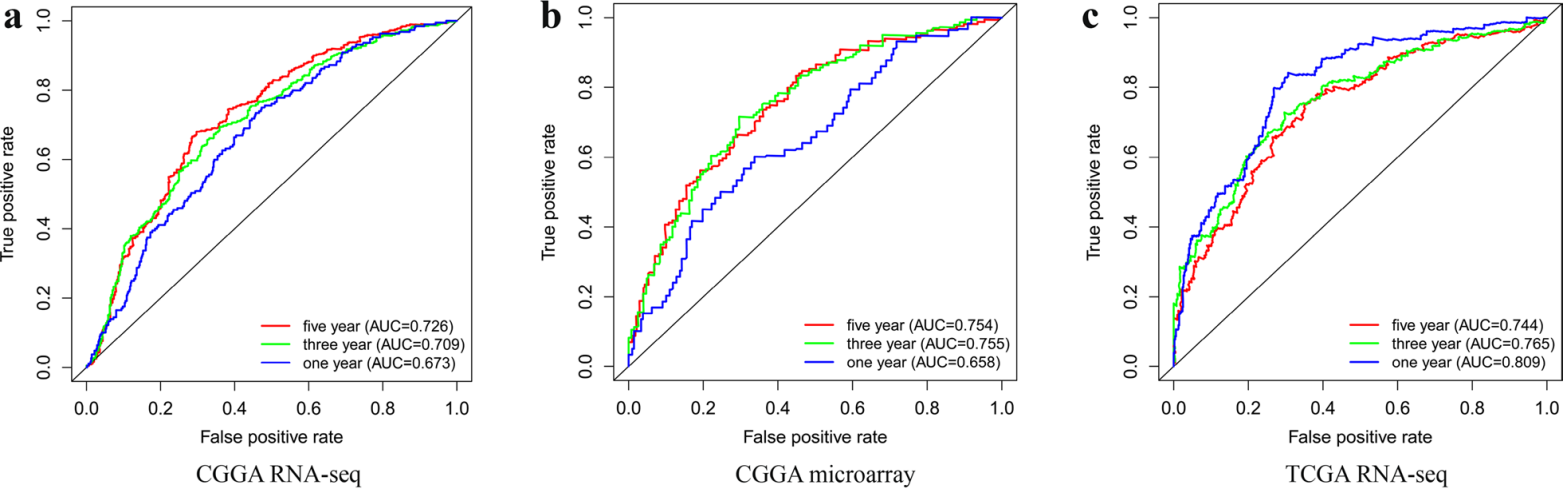
The abnormal high expression of *CTSC* has a relatively reliable predictive value for the survival time of glioma patients.** a**–**c** *CTSC* has a relatively reliable predictive value for 1, 3 and 5 year survival of glioma patients based on CGGA RNA-seq, CGGA microarray and TCGA RNA-seq database

To further explore whether high *CTSC* expression predicts adverse prognosis in patients with glioma, we analyzed clinical samples based from the CGGA RNA-seq, CGGA microarray, and TCGA RNA-seq databases and plotted receiver operating characteristic (ROC) curves (Fig. 5). The area under the curve (AUC) values at 1, 3, and 5 years in all three databases were mostly greater than 0.7, except for the AUC values at 1 year in the CGGA RNA-seq and CGGA microarray databases, which were slightly less than but close to 0.7. These results suggest that high *CTSC* expression level can be used as a predictor of prognosis in patients with glioma.

### **High*CTSC*expression may be an independent risk factor for glioma**

**Fig. 6:**
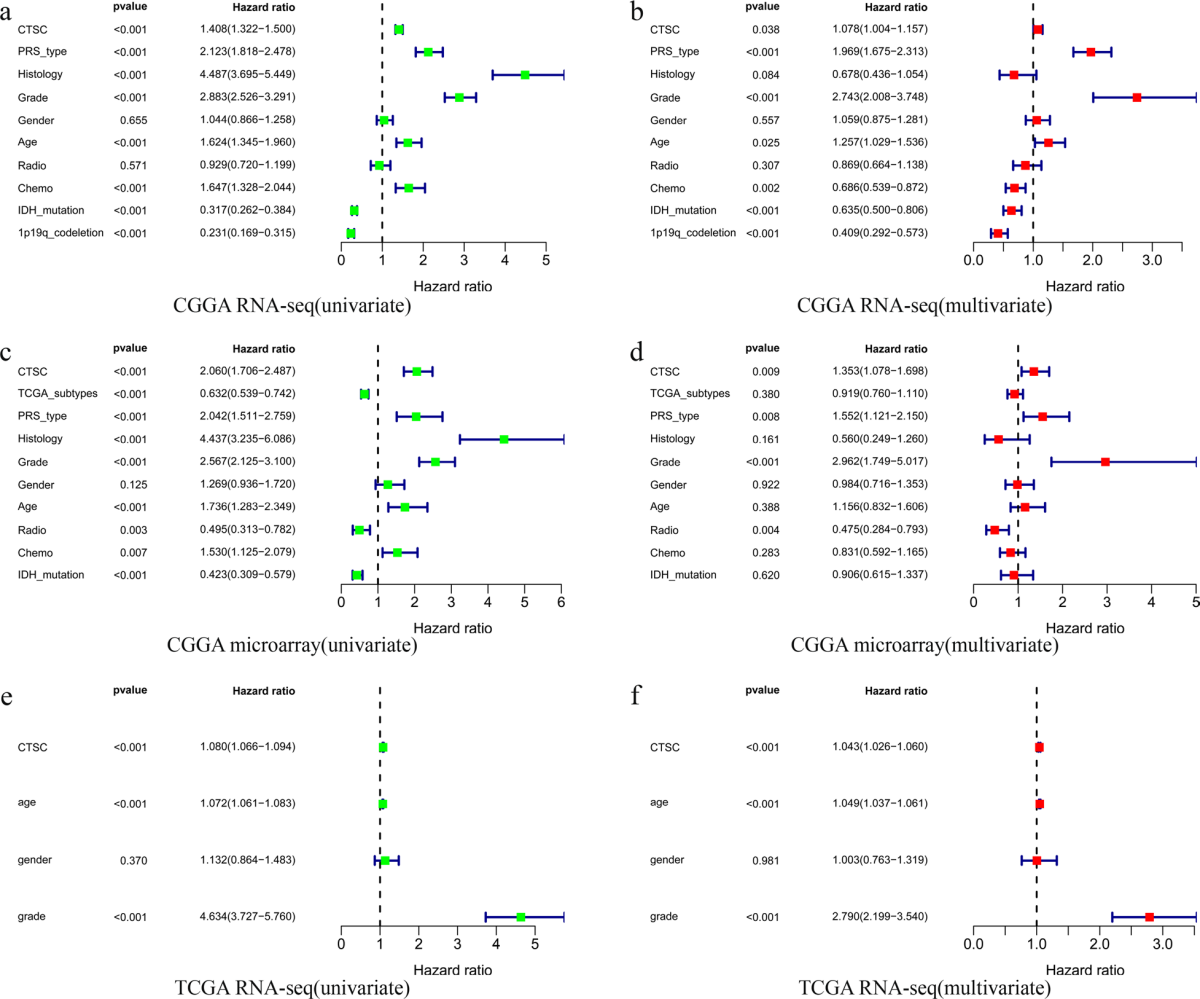
Abnormal high expression of *CTSC* can be an independent risk factor for poor prognosis of glioma patients. **a**, **c**, **e** Univariate analysis based on CGGA RNA-seq, CGGA microarray and TCGA RNA-seq database. **b**, **d**, **f** Multivariate analysis based on CGGA RNA-seq, CGGA microarray and TCGA RNA-seq database

The univariate and multivariate Cox analyses were performed on clinical samples from the CGGA RNA-seq, CGGA microarray, and TCGA RNA-seq databases. The univariate analysis showed that many factors, including *CTSC*, were associated with poor prognosis in patients with glioma (Fig. 6a, c, e). The multivariate analysis showed that high *CTSC* expression might be an independent risk factor for poor prognosis in patients with glioma (Fig. 6b, d, f).

### High expression of ***CTSC*** is associated with multiple clinical features in patients with glioma

**Fig. 7:**
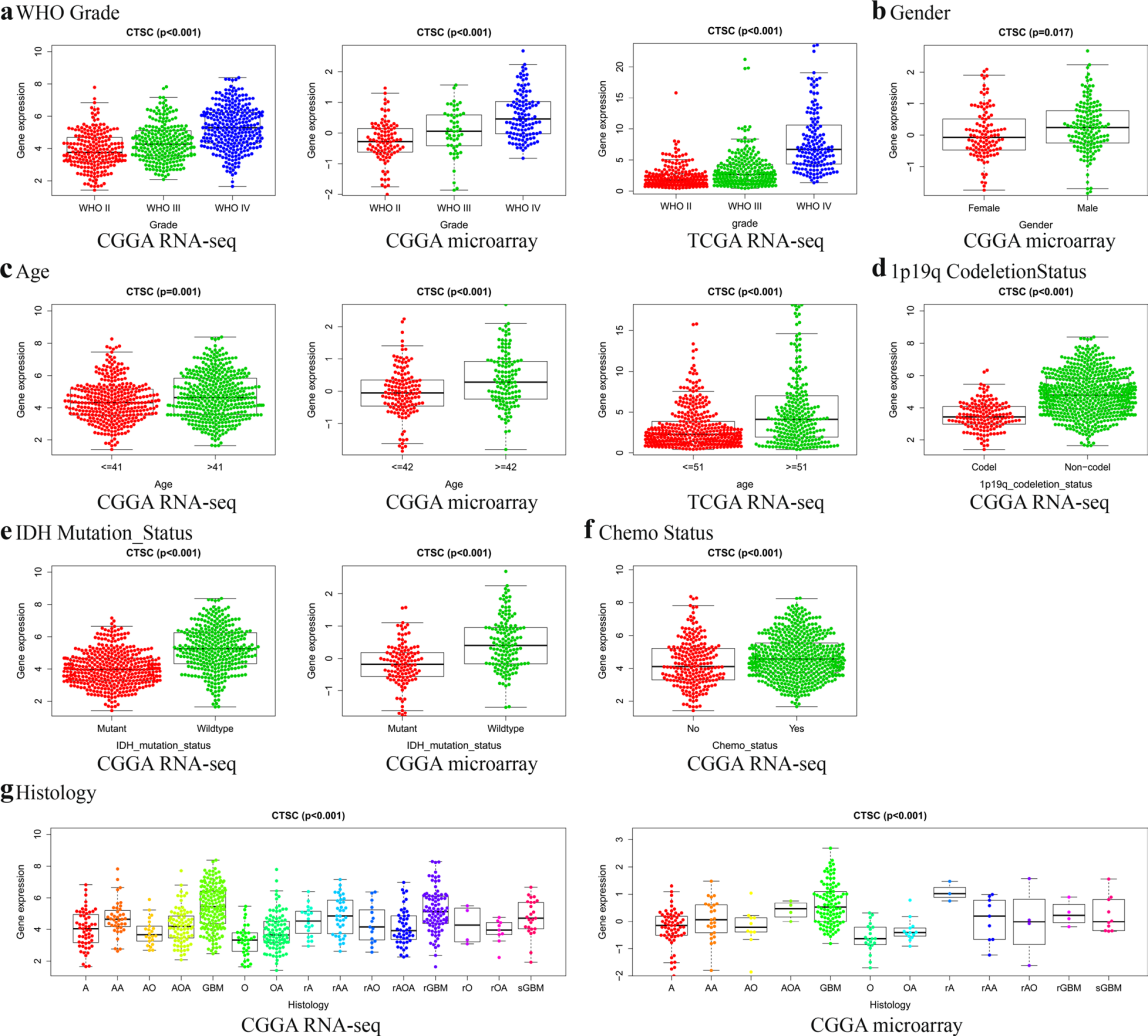
The expression of *CTSC* is associated with various clinical features of glioma. **a** WHO grade. **b** Gender. **c** Age. **d** 1p/19q codeletion status. **e** *IDH* mutation status. **f** Chemo Status. **g** Histology

In our study, clinical samples from the CGGA RNA-seq, CGGA microarray, and TCGA RNA-seq databases were used to reveal the association between *CTSC* and clinical characteristics of patients with glioma. In the CGGA RNA-seq database, high expression of *CTSC* was closely related to clinical features, such as the WHO grade, age, 1p19q co-deletion status, *IDH* mutation status, and histology (Fig. 7a, c–f, g). In the CGGA microarray database, high expression of *CTSC* was closely related to clinical features, such as the WHO grade, sex, age, IDH mutation status, and histology (Fig. 7a–c, e, g). In the TCGA RNA-seq database, high expression of *CTSC* was closely related to the WHO grade and age (Fig. 7a, c). All of the above results were statistically significant (*P* < 0.05). Therefore, we speculate that *CTSC* might participate in the pathological process and malignant progression of glioma.

### GSEA

**Fig. 8:**
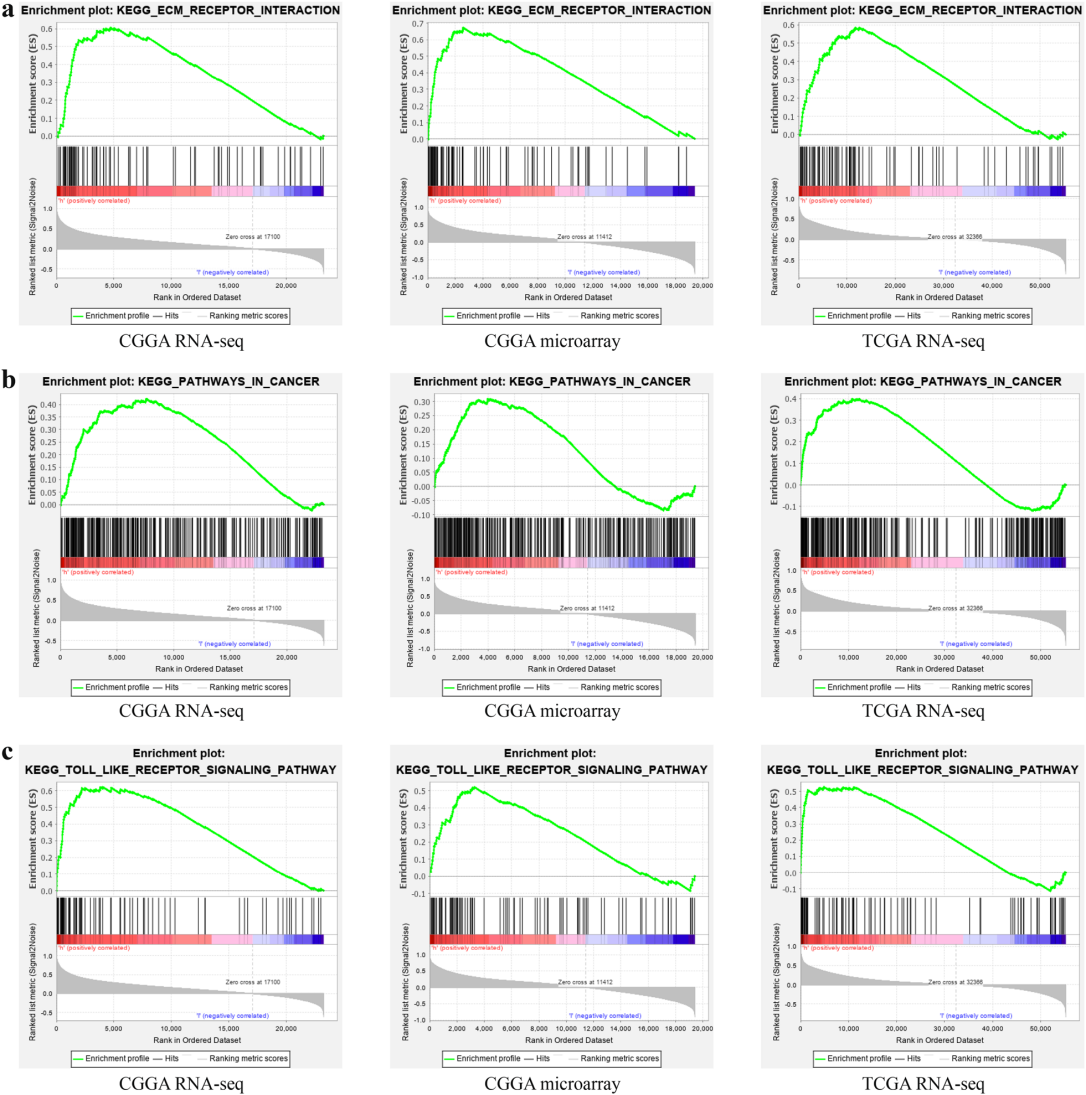
*CTSC* may be involved in the signaling pathway in glioma based on GSEA. **a** ECM receptor interaction signaling pathway. **b** Pathways in cancer signaling pathway. **c** Toll like receptor signaling pathway

To further explore the specific mechanism by which *CTSC* affects the malignant progression of glioma, we used GSEA to identify the signaling pathways in which *CTSC* might participate. We found that *CTSC* participated in the Toll-like receptor signaling pathway, cancer pathways, and ECM receptor interaction signaling pathway (Fig. 8; Table 1), which are all well-known pathways regulating the process of glioma.

**Table 1:** Cell signaling pathway that *CTSC* may be enriched

Gene set name	CGGA RNA–seq			CGGA microarray			TCGA RNA–seq		
	NES	NOM p–val	FDR q–val	NES	NOM p–val	FDR q–val	NES	NOM p–val	FDR q–val
TOLL LIKE RECEPTOR	1.964	0.000	0.003	1.816	0.012	0.028	1.822	0.006	0.018
PATHWAYS IN CANCER	1.616	0.026	0.082	1.412	0.041	0.232	1.592	0.023	0.076
ECM RECEPTOR INTERACTION	1.853	0.014	0.014	2.006	0.002	0.021	1.737	0.021	0.036

*NES* normalized enrichment score, *NOM* nominal, *FDR* false discovery rate. NOM p-value < 0.05 and FDR q-value < 0.25

### Co-expression analysis

**Fig. 9:**
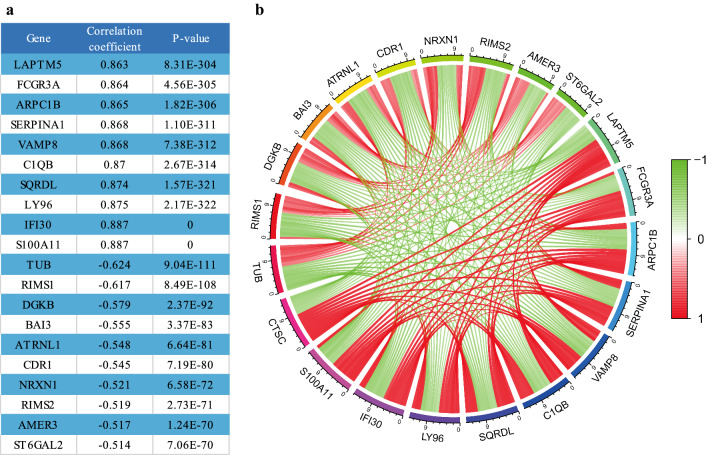
Genes associated with *CTSC* based on co-expression analysis

To verify the previous results, we conducted a co-expression analysis to identify genes, whose expression levels positively or negatively correlated with *CTSC* expression. We used Pearson’s correlation to identify 10 genes that were most synergistically expressed with *CTSC* and 10 genes whose expression was most antagonistic to that of *CTSC*. Detailed information regarding these genes is presented in Fig. 9.

### CMap analysis

**Fig. 10:**
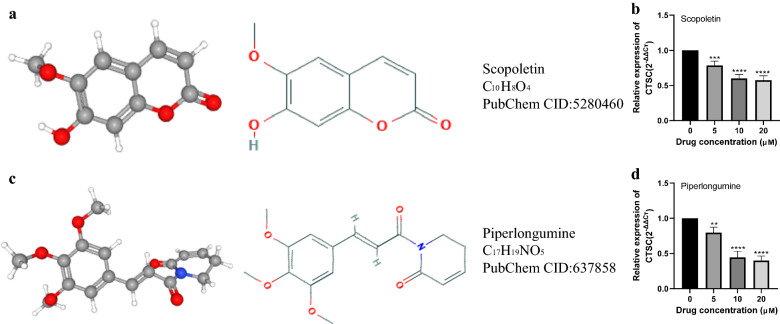
Two small molecular compounds with inhibitory effect on *CTSC* expression based on CMap. **a** Scopoletin’s two-dimensional structure and three-dimensional structure. **b** Expression of CTSC in glioma cells after treatment with different concentrations of scopoletin. **c** Piperlongumine’s two-dimensional structure and three-dimensional structure. **d** Expression of *CTSC* in glioma cells after treatment with piperlongumine at different concentrations. **P < 0.01, ***P < 0.001, ****P < 0.0001

In addition to exploring the involvement of *CTSC* as an oncogene in the occurrence and development of glioma and elucidating the specific mechanism of its action, we conducted CMap analysis to search for small-molecule compounds that can inhibit *CTSC* expression in the hope that these drugs may be used for glioma treatment. In our study, the following two small-molecule compounds were identified: piperlongumine (PL) and scopoletin (SCO). Using the PubChem online tool, we found detailed information such as the name, PubChem CID, molecular formula, 2D, and 3D structure for these small-molecule compounds (Fig. 10a, b; Table 2).

**Table 2:** Screened drugs from CMap

No.	CMap name	Enrichment	*P*
1	Scopoletin	− 0.911	0.01567
2	Piperlongumine	− 0.907	0.01724

Enrichment<− 0.8, *P* < 0.05. *CMap* connectivity map

To verify the above analysis results, the LN-229 cells were treated with PL and SCO at different concentrations. After 8 h treatment, RT-qPCR was used to detect *CTSC* mRNA levels. We found that *CTSC* expression was significantly inhibited by both drugs at concentrations of 5, 10, and 20 µM (*P* < 0.05), with the inhibitory effect of PL being more obvious than that of SCO (Fig. 10c, d), accompanied by the obviously reduced proliferation of glioma cells (data not shown), indicating two novel drugs targeting on *CTSC* for the treatment of glioma.

## Discussion

CTSC is an important acid hydrolase in the lysosomes. In addition to its important physiological functions, CTSC is also involved in the regulation of various pathological mechanisms of cancer. Previous studies have shown that *CTSC* is upregulated in a variety of malignant tumors, including squamous cell carcinoma, pancreatic cancer, and breast cancer [30–32]. Our research is the first to use clinical sample data from multiple databases to comprehensively and systematically analyze and reveal the correlation of *CTSC* expression pattern in glioma, with the aim of identifying a new target marker for the diagnosis and prognosis of this cancer.

In this study, we found increased expression of *CTSC* in glioma based on the data from the GEPIA database. Next, we observed the same results at the molecular and protein levels based on the samples from the GEO and HPA databases. However, whether the high expression of genes has an impact on the malignant progression of glioma is the clear role of oncogenes. Therefore, the relationship between *CTSC* and the prognosis and malignant clinical characteristics of glioma patients still needs further research.

In subsequent experiments, we not only found that abnormally high expression of *CTSC* was associated with significantly shorter survival time of patients with glioma, but showed that it could also be used as an independent risk factor and a valuable prognostic parameter for patients with glioma based on bioinformatics methods. The expression of *CTSC* positively correlated with the grade of glioma and closely associated with the noncoding status of 1p/19q and wild-type *IDH* genotype. Previous studies have shown that the coding status of 1p/19q and mutated *IDH* are associated with higher chemosensitivity and, therefore, are markers of good prognosis in patients with glioma [10, 33]. Hence, we speculated that there was a close correlation between high *CTSC* expression and malignant progression of glioma. However, because the mechanism by which *CTSC* expression affects the malignant process of glioma is unknown, we conducted GSEA to identify the signaling regulatory pathways related to *CTSC* expression. GSEA showed that *CTSC* expression is involved in the ECM receptor interaction pathway, Toll-like receptor signaling pathway, and cancer cell signaling pathways, which are known to promote the malignant progression of various cancers. For example, Zhang et al. found that high expression levels of *ITGA6* and *CD44* in the ECM receptor interaction pathway promote the malignant progression of kidney cancer [34]. Previous studies have found that the Toll-like receptor signaling pathway plays a key role in the immune function of the body and that its abnormal activation affects the pathological process and development of tumors. Toll-like receptors are important for the pathological process and malignant progression of various malignant tumors, including pancreatic cancer, pharyngeal cancer, breast cancer, and head and neck cancer [35–39]. It can be speculated that *CTSC* may promote malignant progression and poor prognosis of glioma through the ECM receptor interaction pathway and Toll-like receptor signaling pathway. In addition, it should be mentioned that pathological development of glioma is not determined by individual biological pathways but by the interaction of multiple cancer pathways and factors. In our study, we focused on the expression of *CTSC* in glioma and its relationship with the prognosis of patients with glioma, but we did not further study the signaling pathways through which high *CTSC* expression affected the malignant process of glioma. However, we believe that our research will provide important ideas and lay a foundation for further studies of the pathological mechanisms of glioma and contribute significantly to its diagnosis and prognosis.

The key role of genes co-expressed with *CTSC* in malignant tumors (including glioma) has also been widely studied. Previous study has shown that the oncogene *LAPTM5* promotes the malignant process of a variety of cancers and its high expression level strongly correlates with the poor prognosis of GBM patients [40]. In addition, *SERPINA1* expression was also found to be higher in malignant high-grade glioma, which correlated with poor prognosis for patients [41]. The correlation between the above-mentioned genes co-expressed with *CTSC* and the degree of glioma malignancy indirectly supports the key role of *CTSC* in the pathological development of glioma. In addition, researchers have also found that *BAI3*, a gene whose expression negatively correlated with that of *CTSC*, was expressed less in glioma than in normal brain tissue [42]. The high expression of *BAI3* indicates a relatively better prognosis for patients with glioma, which also indirectly provides evidence for a positive correlation between high *CTSC* expression and glioma malignancy [42]. Based on the above studies, we indirectly verified the key role of *CTSC* as an oncogene in glioma through the co-expression analysis. However, the ultimate goal of studying tumor markers is to find plausible targets for the treatment of tumors. Therefore, we also performed relevant experiments to identify drugs that could inhibit *CTSC* expression and thereby possibly attenuate glioma growth.

In particular, by using the CMap analysis, we identified two small-molecule compounds, PL and SCO, that inhibited *CTSC* mRNA expression in glioma cells as was confirmed by RT-qPCR. PL, a bioactive alkaloid/amide derived from *Piper longum*, possesses many pharmacological properties, including anti-inflammatory and anti-tumor activity mediated the induction of oxidative stress, and has low toxicity [43, 44]. Several studies have found that PL inhibits the proliferation and migration of glioma cells [45–47]. Furthermore, although PL is cytotoxic against a variety of cancer cells, it shows little toxicity to normal human cells, indicating that it can be a good antitumor agent [48]. Our study confirmed the targeted inhibition of *CTSC* expression in glioma cells by PL, which may be the key mechanism by which PL inhibited the malignant progression of glioma. The antitumor effects of SCO have also been widely studied, although to the best of our knowledge, inhibitory effects of SCO on the malignant progression of glioma have not been previously reported [49]. Our study at the genetic level found that SCO targeted and inhibited *CTSC* expression in glioma, indicating that SCO might be a promising antitumor agent for the treatment of glioma. Because the focus of this study was on the relationship between *CTSC* expression and the malignant phenotype and prognosis of glioma, the anti-tumor effects of the screened small-molecule compounds were not investigated in depth, but we believe our study will provide a new idea for drug development in glioma.

In this study, we used data from public databases to reveal the role of *CTSC* in the malignant progression of glioma and analyzed the possibility of using *CTSC* expression level for glioma prognosis. However, our study had some limitations. The sample data lacked some important clinical information, including chemotherapy drug dosage and surgical methods, which is a common problem in public databases. In addition, compared with glioma samples, the number of normal samples was small, which may have led to bias. Therefore, we conducted RT-qPCR to further verify the results and found that *CTSC* expression was higher in tumor samples and cells than in normal human astrocytes, which once again confirmed the accuracy and reliability of our study.

## Conclusions

Our results confirm that high expression of *CTSC* in gliomas can shorten survival time of patients, and is related to the malignant clinical characteristics of gliomas, which can be used as an independent risk factor of gliomas. The results of this study expand the molecular biological function of *CTSC*, reveal the development mechanism of glioma from a new perspective, and provide a potential biomarker for the prognosis of glioma. Furthermore, the small molecule drugs we identified also enrich the choice of glioma clinical treatment and lay a solid foundation for improving the quality of life of patients with glioma.

## Supplementary Information

**Additional file 1: Table S1**. Characteristics of patientswith glioma based on CGGA RNA-seqdata. **Table S2**. Characteristics of patientswith glioma based on CGGA microarray data. **Table S3**. Characteristics of patientswith glioma based on TCGA RNA-seq data

## Data Availability

The datasets used and/or analysed during the current study are available from the corresponding author upon reasonable request.
